# Understanding genetic and environmental complexity in phenotypic resemblance across relatedness in population-scale data

**DOI:** 10.64898/2026.10.18.759872

**Published:** 2026-10-19

**Authors:** Mariadaria K. Ianni-Ravn, Andy W. Dahl, John Novembre

## Abstract

A fundamental feature of a trait’s variation in a population is how its phenotypic similarity scales with genetic relatedness. Under a strictly additive model of genetic architecture, phenotypic similarity increases linearly with genetic relatedness, with a slope equal to the heritability; this underpins approaches to heritability estimation from sibling IBD regression to Haseman–Elston regression and GREML. Empirically, however, the relationship is not linear for many traits (Kemper et al., 2021). Biobank-scale measurements make fine-grain observations of the phenotypic covariance versus genetic relatedness relationship increasingly feasible, motivating new theory to understand these new empirical observations. Here we evaluate how three processes might introduce non-linearities to the relationship: imperfect tagging of causal variants, assortative mating, and multi-generational shared environments. Through theoretical modeling and simulation, we find each process acts within pedigree classes differently, introducing non-linearities through impacts on the local slope and intercept of phenotypic covariance on genetic relatedness: imperfect tagging lowers the slope among distant relatives, shared environments elevates the intercepts for closer relatives, and assortative mating changes both the slope and intercept. Within these models, we explore a variety of scenarios, finding cases where the local slope of covariance on relatedness values among unrelated pairs versus among siblings can reach nearly three times the raw heritability, and building insight to where heritability estimates are likely to be biased. We then examine these patterns across a range of traits in the UK Biobank, with a supplementary analysis in All of Us. The shape of the relationship varies widely across traits, with profiles exhibiting features found under each of the theoretical mechanisms we explored. Overall, we conclude disentangling these multiple overlaid processes will be necessary to fully understand how phenotypic similarity increases with genetic relatedness in large cohorts.

